# Integration of gene ontology pathways with North American Rheumatoid Arthritis Consortium genome-wide association data via linear modeling

**DOI:** 10.1186/1753-6561-3-s7-s94

**Published:** 2009-12-15

**Authors:** Jérémie JP Lebrec, Tom WJ Huizinga, René EM Toes, Jeanine J Houwing-Duistermaat, Hans C van Houwelingen

**Affiliations:** 1 Department of Medical Statistics and Bioinformatics, Leiden University Medical Center, Leiden, P.O. Box 9604, 2300 RC Leiden, The Netherlands; 2 Department of Rheumatology, Leiden University Medical Center, Leiden, P.O. Box 9604, 2300 RC Leiden, The Netherlands

## Abstract

We describe an empirical Bayesian linear model for integration of functional gene annotation data with genome-wide association data. Using case-control study data from the North American Rheumatoid Arthritis Consortium and gene annotation data from the Gene Ontology, we illustrate how the method can be used to prioritize candidate genes for further investigation.

## Background

By definition, heritable complex traits like rheumatoid arthritis (RA) are caused by several mutations with inherently small effect size. Some of those mutations have low frequency and therefore can not be reliably detected, even in large studies. Nevertheless, it is plausible that causal mutations for a given disease belong to a set of common biological pathways. Such external biological information about gene function is now widely available (e.g., gene ontogeny (GO) [1] and KEGG [2]). Methods for integration of this biological knowledge were first developed by microarray analysts in order to improve power and reproducibility of transcriptome studies. Recently, some attempts [3,4] have been made to transfer this approach into the genomics field. The aim of such data integration is twofold. Firstly, this approach could be used to identify whether some predefined gene sets are over-represented in genome-wide association studies (GWAS) and thus are likely to play a role in disease etiology. Secondly, the available biological information could be used to supplement the candidate disease gene hierarchy provided by the GWAS data. We focus on the second of those two objectives using case-control GWAS data from the North American Rheumatoid Arthritis Consortium (NARAC) provided by Genetic Analysis Workshop 16 (GAW16). Currently existing methods are still in their infancy [4,5] (see Sohns et al. [6] in this issue for an application of the method). We have previously proposed a modeling framework for integration of linkage data [7] with pathway information and have applied it to a linkage scan of the NARAC sample set [8] using 47 GO biological processes known to be involved in rheumatoid arthritis [9]. In this paper, we have now derived a similar model for integration of GWAS signals and gene set or pathway information that will enable researchers to prioritize candidate genes for further investigation.

## Methods

### Data definition

The pathway gene annotation provides information at the gene level. Formally, all *n *human genes may or may not be involved in the *n *pathways considered relevant to the disease of interest. This information is gathered in the *n *× *p *design matrix *n *where *X*_*ij *_= 1, if gene *n *belongs to pathway *n *and *X*_*ij *_= 0, otherwise. In turn, the GWAS signal is measured at the single-nucleotide polymorphism (SNP) level. As in Wang et al. [3], we chose to summarize the GWAS signal at the gene level so as to drastically reduce the dimension of the problem. Each SNP was assigned to the nearest gene (to more than one gene when genes overlap) and was analyzed using the Cochrane-Armitage trend test after application of standard filtering criteria (genotype call rate > 95%, Hardy-Weinberg equilibrium *p*-value > 0.001, minor allele frequency > 0.01, and exclusion of seven individuals with sex classification inconsistent with genetic data). For each gene, the signal at the most significantly associated SNP was selected. Note that the chosen SNP-to-gene annotation strategy may lead to incorrect annotation if a SNP is in linkage disequilibrium with another SNP within a more distant gene or plays a role in the regulation of that gene. The effect size and the related asymptotic variance of the maximal SNP for each gene were then estimated by logistic regression using the number of SNP alleles as a continuous variable in the model. Note that the sign of the effect size was arbitrarily chosen as positive to ensure the incorporation of simple parameters of pathway effects in Model (1). For each gene indexed by *n*, we therefore obtained a GWAS signal denoted *y*_*i *_equal to the positive allelic effect (on the log odds scale) of the maximal SNP mapping to the gene and a related variance  given by the asymptotic estimate.

### A hierarchical model for integration of GWAS signal with pathway information

As defined above, *y*_*i *_is an estimate of the underlying gene effect *μ*_*i*_. We further assume that *y*_*i *_~ *N*(*μ*_*i*_, ) and although the corresponding observations at nearby genes may be correlated, we use a diagonal working covariance. The association signal at the SNP level has mean equal to 0 on the log odds scale if the SNP is unrelated to disease, the *y*_*i *_values have positive expectation because they are taken as the positive log odds effect of the most significant SNP mapped to each gene, and their distribution is skewed and departs from normality. In addition, while it is not entirely clear how  (defined as the variance of a SNP effect) relates to the variance of the *y*_*i *_signal (taken as the maximal signal), it remains closely related to it and is used in our approximate model. In order to introduce pathway effects in the model, we denote by *β*_*j *_the effect of pathway *j *and further assume that given pathway effects, genes act independently and are distributed as

where *μ*_0 _is an overall average gene effect (this parameter includes the positive bias that results from the definition of *y*_*i *_as a positive quantity) and *τ*^2 ^denotes the between-gene variance. Marginally, the summarized GWAS signal *y*_*i *_is thus distributed as

Given *τ*^2^, estimates ,  of *μ*_0_, *β *are given by the normal equations and thus have an explicit form. Maximum-likelihood estimates of all parameters can thus easily be obtained by numerical maximization of the profile likelihood.

### Posterior gene effects

The previous model can be given an (empirical) Bayesian interpretation. The prior gene effects *μ*_*i *_reflecting pathway effects are updated in the light of new GWAS data *y*_*i*_. The posterior gene effects are distributed as

and the standardized posterior gene effects provide an overall ranking for genes. Note that each posterior gene effect is a simple weighted average of the prior gene effect (based on pathway information) and the GWAS signal in the vicinity of the gene. The relative weight of these two sources of information is governed by the ratio /*τ*^2^.

## Results

### Integration of GWAS and pathway information

While we originally intended to use the same 47 GO biological processes derived in Aidinis et al. [9] and used in Lebrec et al. [7], we have restricted our analysis to 27 pathways provided in the c5 MSigDB gene set definition [10] to ensure comparability with other members of our GAW16 working group. We carried out a forward-stepwise regression procedure in Model (2) in order to select the most relevant pathways among the 27 considered. The fitting process is shown in Table 1. Model selection was based on the Akaike information criterion (AIC). Biological processes associated with a positive effect include GO:0009605 (response to external stimulus), GO:0030036 (actin cytoskeleton organization and biogenesis), GO:0006508 (proteolysis), and GO:0009100 (glycoprotein metabolic process). Other negatively associated pathways have genes in common with those four positive pathways and therefore help in refining their effects. For instance, *TP53 *is a component of both GO:0009605 ( = + 0.018) and GO:0006996 ( = - 0.015), while *ABCF1 *is only found in GO:0009605. The corresponding prior gene effects are therefore +0.018 for *ABCF1 *and +0.018 - 0.015 = 0.003 for *TP53*. Table 2 shows the number of genes in each GO set and the number of genes in common between them. We then ranked all genes according to the standardized posterior gene effect computed based upon the distribution in Eq. (3) using the empirical Bayes estimates  and . Genes with the highest standardized posterior gene effects are located in the HLA region on chromosome 6 where *p*-values of association are the most significant. Due to the strong effect of the GWAS signal in that region, any prior effect based on pathway information is overruled by the GWAS signal. It is however possible to tune the relative influence of GWAS signal and pathway effects by increasing the /*τ*^2 ^ratio in the posterior gene effects calculation. We focused on the 1% most highly ranking genes when the tuning is extreme (/*τ*^2 ^was set to 100), i.e., when only pathway effects determine posterior gene effects. Seventeen consensus genes arose (they were defined as being part of both extreme priority lists obtained using the genes ranked in the top 1% when either /*τ*^2 ^is as initially estimated or when /*τ*^2 ^is set to 100): *ABCF1*, *AGER*, *AIF1*, *BACE2*, *C2*, *CAPG*, *CCL1*, *CCL18*, *CD40*, *CDKN1A*, *DOCK2*, *L3MBTL4*, *NEBL*, *NFX1*, *RND3*, *RPS19*, and *TNXB*. The list generated is unlikely to be random because it contains at least four genes reported to be associated with RA. Under the assumption that 100 such genes have already been identified among ≈20,000 human genes, the probability of detecting four or more of those 100 genes in 17 genes drawn at random is ≈10^-6^. It therefore seems that it contributes additional information as compared to the results from GWAS alone. *AIF1 *and *CD40 *were recently confirmed as susceptibility genes for RA [11,12] but *CD40 *(rs1569723, nominal trend test *p*-value ≈ 10^-4^) would not have been prioritized as an RA-associated locus according to the NARAC GWAS data alone.

**Table 1:** Forward-stepwise selection in NARAC data with 27 GO biological processes

					**Pathway effect estimates for consecutive models obtained when adding terms as shown in column 1**										
					
**Model^a^**	**Deviance**	**AIC**	^**b**^									**Selected Model**			

**Intercept**		-65279.5	0.001916	0.16	0.16	0.16	0.16	0.16	0.16	0.16	0.16	**0.16**	0.16	0.16	0.16
**+GO:0009605^c^**	4.27	-65285.99	0.001915		0.017	0.017	0.017	0.018	0.018	0.017	0.017	**0.018**	0.021	0.022	0.018
**+GO:0006996**	2.29	-65288.57	0.001913			-0.010	-0.010	-0.014	-0.014	-0.015	-0.015	**-0.015**	-0.014	-0.014	-0.014
**+GO:0006968**	1.75	-65290.08	0.001911				-0.025	-0.025	-0.024	-0.024	-0.024	**-0.024**	-0.024	-0.024	-0.034
**+GO:0030036**	1.62	-65291.31	0.001909					0.020	0.035	0.035	0.035	**0.035**	0.035	0.035	0.035
**+GO:0007015**	3.07	-65295.45	0.001907						-0.055	-0.055	-0.055	**-0.055**	-0.055	-0.055	-0.055
**+GO:0006508**	1.53	-65296.50	0.001904							0.013	0.013	**0.013**	0.013	0.013	0.013
**+GO:0009100**	0.99	-65296.47	0.001904								0.016	**0.017**	0.017	0.017	0.017
**+GO:0006629**	1.07	**-65296.61**	**0.001903**									**-0.008**	-0.008	-0.008	-0.008
+GO:0006950	0.46	-65295.54	0.001902										-0.005	-0.005	-0.007
+GO:0009617	0.49	-65294.52	0.001902											-0.019	-0.007
+GO:0006952	0.85	-65294.23	0.001902												0.012

^a^GO definitions: GO:0009605, response to external stimulus; GO:0006996, organelle organization and biogenesis; GO:0006968, cellular defense response; GO:0030036, actin cytoskeleton organization and biogenesis GO:0007015, actin filament organization; GO:0006508, proteolysis; GO:0009100, glycoprotein metabolic process; GO:0006629, lipid metabolic process.

^b ^, the between-gene variance estimate

^c ^Bold font indicates elements included in the selected model.

**Table 2:** Number of genes in common between 8 GO biological processes retained in model for NARAC SNP data^a^

Model^b^	GO:0009605	GO:0006996	GO:0006968	GO:0030036	GO:0007015	GO:0006508	GO:0009100	GO:0006629
	0.018	-0.015	-0.024	0.035	-0.055	0.013	0.017	-0.008
GO:0009605	283	15	14	9	1	8	1	13
GO:0006996		424	0	97	23	11	1	10
GO:0006968			49	0	0	0	0	1
GO:0030036				97	23	1	0	1
GO:0007015					23	0	0	0
GO:0006508						166	6	6
GO:0009100							76	11
GO:0006629								279

^a^Row 2 displays the corresponding pathway effect estimate in selected model

^b^GO definitions: GO:0009605, response to external stimulus; GO:0006996, organelle organization and biogenesis; GO:0006968, cellular defense response; GO:0030036, actin cytoskeleton organization and biogenesis GO:0007015, actin filament organization; GO:0006508, proteolysis; GO:0009100, glycoprotein metabolic process; GO:0006629, lipid metabolic process.

## Discussion

Our data and methodology point towards an added value of combining pathway information with GWAS data to possibly assist in the prioritization of candidate genes for further study. However, we resorted to quite a few approximations to cast the problem into the gaussian framework of Model (2). In analogy to the *t*-test, we can realistically hope that the forward stepwise fitting process that we used is robust to the normality assumption. It is beyond the scope of this manuscript to fully assess the robustness of the methodology but the equal variance assumption in Model (1) should especially be challenged. Taking the SNP with maximum association as a response in Model (2) might favor pathways with large genes, as suggested by one reviewer, this bias might be reduced by inclusion of gene size as a predictor in Model (2). This modification of the regression hardly changed the selected model (data not shown) and left top ranking genes unaltered. It is of note that the proportion of between-gene variance explained by the retained model (≈1%) is much smaller than in the case of linkage data (≈50%) [6]. In spite of the fact that SNP data is at a higher resolution than gene-level data, we have chosen a gene-level summary signal due to computational limitations. This may represent a simplistic model that may result in large amount of information being discarded. It will therefore be highly relevant to run this algorithm with SNP-level information for association signals together with pathway information at SNP level to exploit all the available data being generated. Given the wealth of biological information available, it is enticing to use Model (2) with hundreds of candidate pathway predictors. However, the risk of over-fitting would be high. In our opinion, restricting the set of initial candidate pathways to a limited number represents a better approach.

## Conclusion

The empirical Bayesian linear model for GWAS and pathway data derived in this study offers a simple and flexible framework for data integration. Because new tools for candidate gene prioritization are rapidly arising [4,5,13], comparison between methods and their relative usefulness for researchers to prioritize genes is warranted.

## List of abbreviations used

AIC: Akaike information criterion; GAW16: Genetic Analysis Workshop 16; GO: Gene ontogeny; GWAS: Genome-wide association study; NARAC: North American Rheumatoid Arthritis Consortium; RA: Rheumatoid arthritis; SNP: Single-nucleotide polymorphism.

## Competing interests

The authors declare that they have no competing interests.

## Authors' contributions

HCVH conceived of the study and participated in its design. JJD-H helped with the statistical analysis. TWJH and REMT participated in the design of the study. JJPL finalized the statistical analysis and drafted the manuscript. All authors read and approved the final manuscript.
